# Treatment of Hidradenitis Suppurativa Evaluation Study (THESEUS): protocol for a prospective cohort study

**DOI:** 10.1136/bmjopen-2022-060815

**Published:** 2022-04-21

**Authors:** Janine Bates, Helen Stanton, Rebecca Cannings-John, Kim Suzanne Thomas, Paul Leighton, Laura M Howells, Jeremy Rodrigues, Rachel Howes, Fiona Collier, Ceri Harris, Angela Gibbons, Emma Thomas-Jones, Kerenza Hood, John R Ingram

**Affiliations:** 1 Centre for Trials Research, Cardiff University College of Biomedical and Life Sciences, Cardiff, UK; 2 Centre for Trials Research, Cardiff University, Heath Park, Cardiff CF14 4YS, Cardiff University, Cardiff, UK; 3 Cardiff University Centre for Trials Research, Cardiff, UK; 4 Dermatology, University of Nottingham, Nottingham, UK; 5 Division of Primary Care, University of Nottingham, Nottingham, UK; 6 University of Nottingham Centre for Evidence Based Dermatology, Nottingham, UK; 7 Warwick Clinical Trials Unit, University of Warwick, Warwick, UK; 8 Burns and Plastic Surgery, Stoke Mandeville Hospital, Aylesbury, Buckinghamshire, UK; 9 NHS Forth Valley, Stirling, UK; 10 C/O Centre for Trials Research, Cardiff University College of Biomedical and Life Sciences, Cardiff, UK; 11 Centre for Trials Research, Cardiff University, Cardiff, UK; 12 Division of Infection and Immunity, Cardiff University, Cardiff, UK

**Keywords:** DERMATOLOGY, Laser therapy, Surgical dermatology

## Abstract

**Background:**

Hidradenitis suppurativa (HS) is a chronic, painful, inflammatory skin disease with estimates of prevalence in the European population of 1%–2%. Despite being a relatively common condition, the evidence base for management of HS is limited. European and North American management guidelines rely on consensus for many aspects of treatment and within the UK variations in management of HS have been identified. The HS James Lind Alliance Priority Setting Partnership (PSP) published a top 10 list of future HS research priorities including both medical and surgical interventions. The aims of the THESEUS study are to inform the design of future HS randomised controlled trials (RCTs) and to understand how HS treatments are currently used. THESEUS incorporates several HS PSP research priorities, including investigation of oral and surgical treatments. Core outcome domains have been established by the HIdradenitis SuppuraTiva cORe outcomes set International Collaboration (HISTORIC) and THESEUS is designed to validate instruments to measure the domains.

**Methods and analysis:**

The THESEUS study is a prospective observational cohort study. Participants, adults with active HS of any severity, will be asked to select one of five HS treatment options that is appropriate for their HS care. Participants will be allocated to their chosen treatment intervention and followed for a period of up to 12 months. Outcomes will be assessed at 3-monthly intervals using HISTORIC core outcome instruments. Video recordings of the surgical and laser operations will provide informational and training videos for future trials. Nested mixed-methods studies will characterise the interventions in clinical practice, understand facilitators and barriers to recruitment into future HS RCTs and examine patients’ and clinicians’ perspectives on HS treatment choices.

**Trial registration number:**

ISRCTN69985145.

**Strengths and limitations of this study**

- Introduces deroofing technique to the UK and re-tasks laser for hidradenitis suppurativa (HS) (rarely used for HS in UK before).
- Encourages multidisciplinary team working between dermatologists and surgeons and mirrors clinical practice with shared decision-making between patients and clinicians.
- Testing instruments developed by the HIdradenitis SuppuraTiva cORe outcomes set International Collaboration core outcomes set initiative.
- Limitation: Permitting treatment switches means efficacy data are more complex to analyse.
- Limitation: Delivery of conventional surgery was hampered by the COVID-19 pandemic.

## Introduction

### Background and rationale

Hidradenitis suppurativa (HS) is a chronic, inflammatory skin disease with global estimates of prevalence varying between 0.03% and 4% of the population; some of the variance is due to underdiagnosis.^1^ The clinical features of HS are recurrent, painful nodules and abscesses, often leading to skin tunnels and scarring. People with HS have significantly impaired quality of life due to both physical and psychological impact (eg, pain, suppuration of pus, social isolation and work disability).^2–4^

The evidence base for the management of HS is relatively sparse, as highlighted in a 2015 Cochrane review^5^ where only 12 randomised controlled trials (RCTs) with a total of 615 participants were identified. European and North American management guidelines rely on consensus for many of their recommendations as a result.^6–8^ Surveys of UK dermatologists have demonstrated variation in treatment strategies, suggesting there may be undesirable variance in treatment in the UK.^9^

The HS James Lind Alliance Priority Setting Partnership (PSP) published a top 10 list of future HS research priorities including both medical and surgical interventions.^10^ The THESEUS study incorporates several HS PSP priorities, including: what is the most effective and safe group of oral treatments in treating HS (ranked number one priority); what is the impact of HS and the treatments on people with HS (ranked third) and what is the best surgical procedure to perform in treating HS (ranked sixth). Across HS trials, there has been heterogeneity in outcome measurement. However, core outcome domains have now been established by the HIdradenitis SuppuraTiva cORe outcomes set International Collaboration (HISTORIC).^11^ THESEUS will contribute to the validation of HISTORIC outcome measurement instruments (OMIs), providing interpretation data and assessing feasibility.

### Objectives

#### Primary objectives

To inform the design of future HS RCTs and to understand how HS treatments are currently used.

#### Secondary objectives

To determine:

1. Feasibility of recruitment for future RCTs of HS treatments.
2. Choice and characterisation of study interventions, (dose of medication, type of surgical techniques used).
3. Current patient pathways and influences affecting patients’ and clinicians’ treatment choices.
4. Choice, feasibility and responsiveness of OMIs, including evaluation of minimum important difference (MID).
5. Consensus-agreed recommendations for future study designs.

Objectives of nested process evaluation studies and consensus workshop:

1. To characterise and document surgical and laser interventions.
2. To understand patients’ perspectives on treatment choices and the implications of these choices for patients’ lives.
3. To understand barriers to and facilitators for recruitment into future clinical studies of HS treatments.
4. To identify up to three specific research questions suitable for future RCTs.
5. To agree key elements of the design including participants and setting, intervention, comparator and outcomes.

An end of study consensus workshop aims to achieve consensus among key stakeholders over priority research questions and proposed study designs for future research into HS.

### Trial design

The THESEUS study is a non-randomised, prospective observational cohort study, with a nested process evaluation. Patient and public involvement (PPI) partners have been involved in the design and conduct of the study throughout.

## Methods: participants, interventions and outcomes

### Study setting

THESEUS will take place in up to 10 participating secondary care sites within the UK. Sites will be selected to reflect a breadth of specialty areas; these include those that offer a multidisciplinary team (MDT) approach, integrating HS medical and surgical care; those that have experience in HS surgery; and those with a dermatology department that is experienced in HS medical management. Participants will be identified from a search of local patient registers or from routine consultations.

THESEUS study funding commenced on 1 April 2019 and will finish on 30 September 2022.

### Eligibility criteria

Adults presenting to their hospital consultant with a diagnosis of active HS that is not adequately controlled by current treatment, and in whom at least one of the study interventions is appropriate for their care, will be approached to participate in the study.

Participants are eligible for the study if they meet all the following inclusion criteria and where none of the exclusion criteria apply:

#### Inclusion criteria

- Adults at least 18 years old with active HS of any severity.
- Diagnosis meets disease definition: a lifetime history of at least five flexural skin boils or two flexural skin boils in last 6 months and confirmed by recruiting clinician with experience of HS care.
- HS is not adequately controlled by current treatment.
- At least one of the five study interventions is appropriate for the participant’s care.

#### Exclusion criteria

- Unable or unwilling to give informed consent.
- Pregnancy or breast feeding.
- Not fluent in English, (questionnaires are only validated in English).

### Who will take informed consent?

Consent will be taken by suitably qualified Principle Investigators (PIs), coinvestigators and research nurses. The recruitment appointment will be carried out face–face for patients who are not known to the recruiter. However, for those patients who are already known to the study team, eligibility, consent and the baseline appointment can be performed remotely, if necessary, without the need for the patient to attend the hospital clinic. The option for remote recruitment was introduced via a protocol amendment as a result of the COVID-19 pandemic.

The consent form will have an option to approve being contacted about taking part in a study interview or joining an end-of-study consensus workshop. Prior to the interview, consent will be taken over the telephone by a study researcher; this process will be audio recorded. Participants who agree to attend the consensus workshop will not be asked formally for their consent, as their attendance will provide implied consent.

### PPI statement

Our PPI partners were involved as co-applicants on the THESEUS study grant and attend regular study management group meetings where they have input on study design (box 1).

Box 1 **Patient and public involvements (PPIs) contribution to the study design**

**PPI and contribution to the study:**

- Suggested to avoid chlorhexidine antiseptic washes as an intervention as this might perpetuate the concept of hidradenitis suppurativa being a condition of personal hygiene that contributes to further stigma.
- When discussing remote follow-up processes our PPIs highlighted it was key to offer participants options, that is, either video call or telephone with photographs of lesions provided or patient self-reporting their lesions as there would be different level of comfort felt by the participants.
- Suggested the addition of an ‘Opt Out’ message for the daily pain score text messages.
- PPIs suggested creating a specific deroofing video for patients (2600 views) as well as healthcare professionals (557 000 views) that avoids surgical jargon.
- Contributed to the planning of the consensus workshop, suggesting that at least one preworkshop meeting be held for patients in order to provide them with background information, including the aims of the workshop and how patients can contribute. Also, to offer the option of remote attendance for those unable to travel to the workshop.

### Interventions

#### Explanation for the choice of comparators

The choice of interventions that will be offered to patients in this study is informed by surveys completed by patients with HS (n=358), dermatologists (n=57) and plastic and general surgeons (n=225 responses analysed to date).^9^ We obtained PPI input through face–face discussion groups involving three people with HS and a partner of someone with HS. This PPI group recommended key changes to proposed study plans which have informed the current protocol (box 1). From these surveys and PPI feedback, five treatments were selected for the THESEUS study.

#### Intervention description

Our study design allows centres to offer standard care, where the clinician and patient feels this is most appropriate, as well as the opportunity to offer treatments that are less commonly used in the UK. Laser treatment and deroofing of skin tunnels are not standard treatments in the UK, but they are both included in the summary recommendations of the European HS guidelines.^12^ As the study is observational in design, normal National Health Service (NHS) waiting lists apply.

Participants can choose one of five treatment options, depending on their suitability as assessed by their clinician and on the availability of the options at each recruiting centre. Each site is required to provide at least four of the five interventions:

1. Oral doxycycline 200 mg one time per day for 6 months, with the option to continue for up to a further 6 months.
2. Oral clindamycin and rifampicin both 300 mg two times per day as a combined course for 10 weeks, with the option to continue up to 6 months.
3. Laser treatment using nd-YAG laser, (skin types 2–6) or alexandrite/diode laser, (skin types 1–3) administered on four occasions each 1 month apart.
4. Deroofing of skin tunnels using electrocautery, (optimal protocol to be determined by UK experts and provided to centres as a training package including a video). It is expected that by following this training video, healthcare professionals including dermatologists and plastic surgeons will be able to perform the intervention. The procedure is carried out under local anaesthetic, repeated during the next 6 months if necessary. The total area treated at one time is limited by the volume of local anaesthetic needed and expected degree of impairment of activities of daily living during recovery. A training video was produced as part of the THESEUS study and so far has been viewed more than half a million times: https://www.youtube.com/watch?v=ftizgrBMzok.^13^
5. Conventional surgery, (narrow or wide excision, with a range of closure methods depending on the preference of the clinician), with the option to receive another intervention or combination of interventions 6 months after the surgery and for up to a further 6 months.

Following the initial 6 months of the chosen intervention, participants have the choice to continue with the intervention or to switch to another intervention or combination of interventions.

### Criteria for discontinuing or modifying allocated interventions

Participants opting for an intervention with a substantial delay before receipt of the intervention, can choose to have another intervention during their wait time.

Participants are asked to stay on their chosen intervention for 6 months, after which they can either continue or switch to another intervention.

Participants who switch interventions during the first 6 months will be followed-up and the reason for the switch will be recorded.

### Relevant concomitant care permitted or prohibited during the trial

All concomitant care interventions are permitted at baseline because the study is observing usual care processes and the need for any additional concomitant care during the study will be documented.

### Provisions for post-trial care

Once a participant has completed the study period, they will revert back to regular NHS care. It is intended that centres offering deroofing may continue to provide this following the study, using the equipment provided by THESEUS and their experience of the procedure gained during the study.

### Outcomes

Primary outcomes measure(s):

- Proportion of participants who are eligible, and hypothetically willing, to use the different treatment options at baseline.

Secondary outcomes measure(s):

- Proportion of participants choosing each of the study interventions, with reasons for their choices.
- Proportion of participants who switch treatments within the first 6 months of treatment, with reasons for switch.
- Treatment fidelity over 6 months.
- Loss to follow-up rates over 12 months.
- Efficacy outcome estimates after 6 months of follow-up to contribute to OMI validation and to inform the end-of-study workshop regarding future RCT designs. Instruments include: lesion counts, HS Quality of Life Questionnaire (HiSQOL), Dermatology Life Quality Index (DLQI), Pain Numerical Rating Scale (NRS), Fatigue Questionnaire, Patient Global Assessment.

#### Sample size

With a sample size of 150, we will be able to estimate the proportion of participants who are hypothetically willing and eligible to be randomised in a clinical study to within a 95% CI of ±7%. We also wish to identify the case mix of patients for each of the possible treatment options. From our patient survey, the least favourable treatment option was minor surgical operations, (13%), which would include deroofing of skin tunnels. One hundred and fifty patients will provide us with 20 patients opting for this non-medical intervention, which is sufficient to explore delivery in the IDEAL 2b evaluation.

### Recruitment

The study team will work closely with the UK Dermatology Clinical Trials Network to publicise the study, provide study updates, facilitate the recruitment of additional sites, if needed while keeping current sites updated on recruitment progress against our target sample size.

The Cardiff Centre for Trials Research (CTR) study team will be in close contact with all recruiting sites, providing a regular newsletter highlighting current recruitment rates, any changes to the recruitment period and other issues that sites need to be aware of.

### Data collection and management

#### Plans for assessment and collection of outcomes

##### Baseline data collection

The THESEUS study will use a bespoke Structured Query Language (SQL) database for data capture. During the baseline appointment the clinician will capture data, as outlined in the schedule of interventions and assessments (table 1), using the baseline case report form, (CRF). Current medical management of their HS will be recorded, including any concomitant medications and interventions received in the last 12 months. The clinician will record the participants’ clinical examination findings, (regions of the body affected by HS, number of inflammatory lesions and the presence and extent of skin tunnels) and determine their Hurley stage^14^ for each region.

**Table 1:** Schedule of interventions and assessments

Review number	−1	0	1	2	3	4
Planned month	−1	Baseline	3	6	9	12
Screening logs	X	X				
Eligibility assessment	X	X				
Demographics and consent		X				
Clinical examination including Hurley stage*		X	X	X	X	X
Interventions for which participant is potentially eligible		X				
Intervention received, with reasons for choice (including treatments switched after baseline)		X	X	X	X	X
**Outcomes**						
HS Quality of Life Questionnaire		X	X	X	X	X
Dermatology Life Quality Index)		X	X	X	X	X
EQ5D-5L questionnaire		X	X	X	X	X
Pain Visual Analogue Scale (NRS)		X	X	X	X	X
Pain score (via text message)		twelve weeks from start of intervention				
Need for dressings		X	X	X	X	X
Fatigue Questionnaire		X	X	X	X	X
Patient Global Assessment		X	X	X	X	X
Anchor question for change in severity			X	X	X	X
Flares		X	X	X	X	X
Assessment of HS physical signs		X	X	X	X	X
Adverse effects of study treatment			X	X	X	X
Treatment fidelity			X	X	X	X
End of study questionnaire (participants and clinicians)						X
Surgeon questionnaires/pro forma		After each surgery				
Structured interview (subset of participants)		Single interview				
Consensus workshop (subset of participants, clinicians and researchers)		Single workshop				

*Hurley stage is specific for each affected region and provides a baseline classification of disease severity in which sporadic lesions not leaving scarring is classed as stage 1, (mild), multiple widely spaced lesions leaving scarring is classed as stage 2, (moderate) and multiple lesions coalescing into inflammatory plaques is classed as stage 3, (severe).

EQ5D-5L, European Quality of Life-5 dimensions; HS, hidradenitis suppurativa; NRS, Numerical Rating Scale.

At the baseline appointment participants will be asked to self-complete the DLQI,^15^ HiSQOL questionnaire,^16^ European Quality of Life-5 dimensions questionnaire,^17^ Fatigue Severity Scale^18^ and a Patient Global Assessment.^19^ Participants will also provide a self-reported NRS pain score, an assessment of skin fluid drainage, their use of dressings to cover wounds, (or soak up blood, pus or other fluid), and the number of flares of HS experienced by the participant in the last month. Participants will complete a one-off CRF asking whether they would hypothetically be willing to receive each of the THESEUS interventions if they were eligible and if the option was available at their treating hospital.

The baseline appointment will conclude with the completion of the baseline intervention CRF: the clinician and the participant together will discuss which intervention(s) they are eligible to receive and, of those options, the participant’s final intervention choice, (primary outcome).

##### Follow-up data collection

Follow-up reviews will be conducted over the telephone or at the outpatient clinic in person at 3 months, 6 months, 9 months and up to 12 months after the baseline appointment. The option for remote telephone follow-up was introduced via a protocol amendment, due to the COVID-19 pandemic and the subsequent restrictions on face-to-face appointments. Follow-up reviews are not anchored to treatment schedules and are conducted regardless of whether the participant has received their THESEUS intervention yet.

The clinical examination will be repeated at each follow-up review and recorded by the clinician. The measures completed at the baseline appointment, with the addition of a change in disease severity anchor measure, will be completed by the participant at each follow-up review.

The clinician will document which treatments or procedures the participant has received at each follow-up review using the intervention CRF. Clinicians will use this CRF to record intervention choice changes and any additions to the participant’s treatment regimen. An adverse event notification CRF is available at each review should the participant report any study-related adverse effects.

##### Pain score text messages

The THESEUS study is testing the feasibility of collecting participants’ daily pain NRS scores via short message service text messages. The text messages are triggered by the addition of specific data items, (depending on the chosen intervention) being added into the baseline intervention CRF within the SQL database.

The text messages are sent by Esendex; a telecommunications service provider. This is an automated process whereby Esendex is instructed to send the same message to the participant asking about the participant’s current pain magnitude due to their HS, daily for up to 12 weeks from the day the intervention was commenced. The text message is sent to the participants at the same time each day, (06:00). The participant has until 02:00 the following day to respond to provide their pain score for that day.

Participants are sent the following text message:

‘Hello. This a text message from the THESEUS study. Please indicate the level of pain you are CURRENTLY experiencing due to your HS. The scale is from 0 to 10. ‘0’ means no pain and ‘10’ means pain as severe as it could be. You have until 02.00 am tomorrow morning to return today’s pain score. If you no longer wish to receive these messages, please text STOP to [THESEUS telephone number].’ ‘Copyright of Cardiff University and HISTORIC.’

##### Nested qualitative studies

###### Study 1

On each occasion where conventional surgery, deroofing or laser intervention is used, the operator will be asked to complete a questionnaire which details the technique used, duration of procedure, adaptations to the procedure and resources used. Up to three centres will be purposefully sampled to video record a small sample of laser and surgical procedures to help document variation in practice and produce materials demonstrating best practice.

###### Study 2

A subset of study participants (approximately 50 participants) who agree to take part in a telephone interview will be asked about their experiences of taking part in THESEUS. This will include factors influencing their choice of intervention and facilitators and obstacles to taking part in HS clinical trials. Purposive sampling of cohort participants will ensure a spread of procedural interventions, (conventional surgery, deroofing and laser treatments) and those opting for drug treatments. Sampling will also ensure diversity in age, gender, ethnicity, HS severity and geographical location. Interviews with at least one clinician at each recruiting site will also be undertaken to receive feedback on taking part in THESEUS from a healthcare professional/researcher perspective.

###### Study 3

All cohort participants and recruiting clinicians will be asked to complete an end of study questionnaire, covering satisfaction with treatments, barriers to and facilitators for participating in HS research, in addition to influences on treatment choice.

##### End of study consensus workshop

The study results will be used to inform a series of multistakeholder workshops, conducted both virtually and face-to-face. The workshop will consider the design and interventions to include in future HS RCTs.

#### Plans to promote participant retention and complete follow-up

The severity of the COVID-19 pandemic in the UK in March 2020, resulted in a national lockdown. Face-to-face appointments for patients became difficult; as a result, the option for remote recruitment and remote follow-ups was implemented in order to improve recruitment and follow-up rates. For participants where remote recruitment or remote follow-up is appropriate, patients have the option of: self-reporting their lesions, providing the site with still photographs, or taking part in a remote video consultation for a clinician to assess their lesions. Video consultations involving assessment of intimate skin regions will be conducted with a chaperone present using a secure NHS approved digital interface.

### Data management

#### Data collection

Quantitative data will be captured electronically and stored within a custom-built SQL database developed within the CTR. Paper copies of CRFs are available if required. Paper CRFs will not be returned to the CTR except in the case of archiving Investigator Site Files.

Data collection can be undertaken by a suitably qualified and experienced clinician, (nurse, doctor or surgeon) employed at the recruiting NHS site.

Qualitative interviews with participants and clinicians will be undertaken by a member of the research team. Interviews will be recorded, transcribed verbatim and stored on a secure access restricted platform at the University of Nottingham.

Participants will be identified by a unique study identification number (PID).

#### Management of text message data

Text message responses from participants will be held in an Application Programming Interface (API) built by Esendex; a telecommunications service provider. A command from the THESEUS SQL database will retrieve the text message data and associated metadata from the Esendex API on a daily basis.

#### Data quality

During data entry validation checks will automatically occur, as validations have been written into the database system.

#### Security

Access to all data, whether paper or electronic, will be restricted to delegated site staff and researchers at the CTR.

#### Confidentiality

A data management plan was developed prior to recruitment commencing.

CTR and study site delegation logs will record who is permitted to enter data and access data held in the THESEUS database. Staff added to the site delegation log will have access to research data relating to participants at their site only. Training on data safety and security will be delivered to local investigators and staff prior to recruitment at site.

#### Personal and identifiable data

Personal and identifiable data about the participant is collected at site. Participant consent is taken at site on a paper form and will contain the participant’s name. Paper-based consent forms will be stored separately to any paper-based clinical data in a locked cabinet. All hard copies of participant data will be stored in locked cabinets.

The THESEUS registration form is an online form which is used to collect participant contact details among other data. Participant registration data are entered directly onto the THESEUS SQL database. This information will be restricted and can only be accessed by using a unique username and password.

#### Electronic data security

The THESEUS SQL database may be accessed remotely via a web interface. The database is held on a secure Cardiff University server and a secure login is required for access. Researchers at recruiting sites can use any device with an internet connection to add data to the database. Data will not be stored offline on any devices. If no internet connection is available, data collection will be done on paper and then transferred on to the database at a later date.

Participants’ mobile phone numbers for the pain score text messages are accessed automatically by Esendex from the participant registration form held in the THESEUS SQL database. The pain NRS score responses from the text messages are imported back into the THESEUS SQL database and linked to the participants’ unique PID. Both the THESEUS Esendex account and the Esendex API can only be accessed by using a unique username and password.

#### Transferring data securely

The CTR will receive a scanned copy of the consent form from the NHS sites participating in the study. Scanned copies of consent forms will be transferred to the THESEUS email account using Cardiff FastFile, a secure file transfer platform.

With participant consent, their contact details will be shared with qualitative researchers based at Nottingham University to enable participants from the main study to be contacted to take part in a qualitative interview. This data will be shared as and when it is needed using a password protected Excel spreadsheet and transferred using Cardiff FastFile.

### Statistical methods

#### Statistical methods for primary and secondary outcomes

We will describe the eligibility and recruitment of participants into the study, their willingness to use the different intervention options at baseline, and their final intervention choice alongside their reasons. The group membership of the final intervention choice will be described using summary statistics using data collected at baseline, (including demographics, clinical history, severity of HS) to determine the drivers of treatment choice. We will also examine patients’ willingness to receive each treatment and their ranking of the treatments, (participant preference), and the clinicians’ assessment of their eligibility. We will describe the number and characteristics of individuals not eligible for each treatment option to understand clinicians’ treatment choices and why individuals were not suitable, to inform a future trial’s eligibility criteria and target group.

Over the study period, we will report whether patients continue with and adhere to their chosen intervention or whether they switch to alternative HS interventions during the study period. Within an intervention option and where there is variation in fidelity, we will explore the characteristics of non-adherence. Where a patient switches intervention, we will report the reasons for this and explore the characteristics of individuals that switch, (including intervention type, skin sites and other baseline demographics). Rates of loss to follow-up over the study period will be reported.

Clinical outcomes will be described using proportions, mean, (SD) or median, (25th–75th centiles) at each time point. (baseline, 3 and 6 months). Effect over time, baseline to 6 months, will be estimated for each efficacy outcome for each group with 95% CIs. Potential confounders of outcome will be assessed using regression methods but as this study is a feasibility study and not powered to detect differences between arms, p values will not be presented. We will estimate the clustering of outcomes within recruitment sites using the intracluster correlation coefficient, (ICC) and compare this ICC estimate with estimates from other relevant large RCTs.

For validation of core outcome instruments, global assessment will be used as our anchor tool to allow estimation of the MID. This will be used to dichotomise outcomes into those with clinically meaningful change and those without clinically meaningful change, (stable or worse). Receiver operating characteristic curves will be generated, and the area under the curve calculated as a measure of the outcome’s ability to detect clinically relevant change. Youden’s J index will be used to determine the minimal important change, and the MID will be calculated as the difference between the mean change in the improved and stable subgroups.^17^

#### Interim analyses

No interim analyses will be performed.

#### Methods for additional analyses (eg, subgroup analyses)

The outcomes will not undergo traditional statistical testing and not powered to detect differences between arms.

Nested study analysis:

##### Study 1

Completed pro formas will be reviewed by the study team to characterise each procedure and to chart how the procedure is being used in a centre. Characterisation will summarise key features of each procedure, such as surgical margins used, closure techniques, types of dressings and so on. Following the characterisation of each procedure an expert panel will review the video recordings to identify which, (in whole or part), typify best practice.

##### Study 2

All data will be charted to a predefined thematic framework^15 16^ which will include matrices for HS treatment, (to include reasons for treatment choice and satisfaction with treatment) and HS research, (to include reflections on current study and recommendations for future research). Charted data will be synthesised, and themes and subthemes interpreted and summarised.

##### Study 3

All data will be charted to the predefined thematic framework used in STUDY 2. Charted data will be synthesised with data from STUDY 2 and themes and subthemes interpreted and summarised.

### Oversight and monitoring

#### Composition of the coordinating centre and study steering committee

Cardiff University is acting act as sponsor for the study, and the CTR at Cardiff University will manage the study. The study will be conducted in accordance with the Research Governance Framework for health and social care, principles of Good Clinical Practice (GCP), General Data Protection Regulation (GDPR) and CTR standard operating procedures.

A THESEUS study management group (SMG) will oversee study design, study centre recruitment study management, study logistics and cost management, study methods, statistical data analysis and publication. The SMG will comprise the chief investigator (CI) and THESEUS grant co-applicants, including our patient representatives, together with the study manager, data manager, statistician and administrator. The Study Manager will be responsible for running the study and will be accountable to the CI.

As a non-randomised, prospective cohort study, a joint Study Steering/Data Monitoring Committee (SS/DM-C) will provide overall study supervision. The role of the SS/DM-C will be to provide overall supervision of the trial on behalf of the National Institute of Health Research. In particular, the SS/DM-C will focus on progress of the trial, adherence to the protocol, participant safety and consideration of new information. There will be four independent members: a chairperson experienced in the conduct of clinical trials, an academic, a biostatistician and a patient representative. The CI will attend all meetings, accompanied by the study manager and other SMG/study staff as appropriate.

Ethics and dissemination

The Wales Research Ethics Committee (REC) 4 provided ethical approval for THESEUS on 26 September 2019 (REC reference 19/WA/0263). Results will be published in international peer-reviewed journals and summaries provided to the funder, participating sites and to study participants, including a plain language summary hosted on the THESEUS study website and promoted via social media. The data sets used and/or analysed during the current study are available from the corresponding author on reasonable request but which would require additional processing to ensure confidentiality.

#### Adverse event reporting and harms

Safety reporting within this study will follow usual care pathways, as THESEUS is an observational study. Investigators will follow their usual processes of reporting adverse events, for example, yellow card reporting when required. There is no requirement to notify the research team in any expedited way of any adverse events.

The research team will collect data on adverse effects that occur during the treatment and follow-up stages of the study via routine data collection at the scheduled follow-up appointments and these will be reported to the REC in an annual progress report.

## Discussion

The THESEUS study will document how HS treatments are currently being used in the UK and will provide vital information to answer the questions that were identified as priority areas for research by HS patients and treating clinicians. Data from THESEUS is intended to inform the design of future HS RCTs. In addition, THESEUS will determine the most suitable outcome measure instruments for future trials and develop improved understanding of patients’ decision-making and experience of treatments.

The THESEUS protocol was modified to take account of the COVID-19 pandemic, for example, by permitting remote assessment of participants. One advantage is that lessons learnt from THESEUS regarding remote assessment may improve the design of future RCTs in this regard. Feasibility of daily text message assessment of pain NRS scores will be important in determining if this is a feasible method of assessing pain in HS.

### Trial status

Protocol V.4.0, 09 December 2021.

Date of first recruit: 18 February 2020.

## Supplementary Material

Reviewer comments
