# Supplementary material for: Treatment of Hidradenitis Suppurativa Evaluation Study (THESEUS): protocol for a prospective cohort study
Source: BMJ Open. 2022 Apr 21;12(4):e060815. doi: 10.1136/bmjopen-2022-060815 (PMC9024265; doi:10.1136/bmjopen-2022-060815)
Supplement: Reviewer comments [file bmjopen-2022-060815.reviewer_comments.pdf]

PEER REVIEW HISTORY

BMJ Open publishes all reviews undertaken for accepted manuscripts. Reviewers are asked to complete a checklist review form (**http://bmjopen.bmj.com/site/about/resources/checklist.pdf**) and are provided with free text boxes to elaborate on their assessment. These free text comments are reproduced below.

ARTICLE DETAILS

**TITLE (PROVISIONAL)**	Treatment of Hidradenitis Suppurativa Evaluation Study (THESEUS): protocol for a prospective cohort study
**AUTHORS**	Bates, Janine; Stanton, Helen; Cannings-John, Rebecca; Thomas, Kim; Leighton, Paul; Howells, Laura; Rodrigues, Jeremy; Howes, Rachel; Collier, Fiona; Harris, Ceri; Gibbons, Angela; Thomas-Jones, Emma; Hood, Kerenza; Ingram, John

This article was not externally reviewed at BMJ Open. Protocols that have been independently assessed prior to submission to BMJ Open are usually fast-tracked to publication on the grounds that further substantial changes will not be possible. This independent assessment will usually be external, independent review for both a substantial grant award from a non-commercial or government funder and ethics approval.
